# A distributed code for color in natural scenes derived from center-surround filtered cone signals

**DOI:** 10.3389/fpsyg.2013.00661

**Published:** 2013-09-27

**Authors:** Christian J. Kellner, Thomas Wachtler

**Affiliations:** ^1^Department of Biology II, Ludwig-Maximilians-Universität MünchenMartinsried, Germany; ^2^Graduate School of Systemic Neurosciences, Ludwig-Maximilians-Universität MünchenMartinsried, Germany; ^3^Bernstein Center for Computational Neuroscience, Ludwig-Maximilians-Universität MünchenMartinsried, Germany

**Keywords:** color vision, visual cortex, sparse coding, natural image statistics, population code, efficient coding

## Abstract

In the retina of trichromatic primates, chromatic information is encoded in an opponent fashion and transmitted to the lateral geniculate nucleus (LGN) and visual cortex via parallel pathways. Chromatic selectivities of neurons in the LGN form two separate clusters, corresponding to two classes of cone opponency. In the visual cortex, however, the chromatic selectivities are more distributed, which is in accordance with a population code for color. Previous studies of cone signals in natural scenes typically found opponent codes with chromatic selectivities corresponding to two directions in color space. Here we investigated how the non-linear spatio-chromatic filtering in the retina influences the encoding of color signals. Cone signals were derived from hyper-spectral images of natural scenes and preprocessed by center-surround filtering and rectification, resulting in parallel ON and OFF channels. Independent Component Analysis (ICA) on these signals yielded a highly sparse code with basis functions that showed spatio-chromatic selectivities. In contrast to previous analyses of linear transformations of cone signals, chromatic selectivities were not restricted to two main chromatic axes, but were more continuously distributed in color space, similar to the population code of color in the early visual cortex. Our results indicate that spatio-chromatic processing in the retina leads to a more distributed and more efficient code for natural scenes.

## 1. Introduction

In the retina of trichromatic primates, spatio-chromatic processing of signals from long (L), medium (M), and short (S) wavelength selective cones by ON and OFF bipolar and ganglion cells with center-surround receptive fields leads to parallel pathways that carry both spatial and chromatic information to the lateral geniculate nucleus (LGN) and visual cortex (Lee, 2011). Chromatic signals are carried in pathways encoding differences between S cone signals and the signals of L and M cones, or differences between L and M cone signals, respectively (Mollon, 1989; Dacey, 2000; Solomon and Lennie, 2007; Lee et al., 2010). While the first, phylogenetically older pathway has low spatial selectivity and is thought to be specifically concerned with color information, the second pathway carries both spatial and chromatic information (Boycott and Wässle, 1999; Martin et al., 2011). Midget retinal ganglion cells have spatially center-surround receptive fields and in the fovea achieve their color opponency by antagonistic processing of signals from a single cone in the center and several cones in the surround of their receptive field. There is evidence for functional cone-type specificity beyond that arising from a single-cone center, but different studies have arrived at different conclusions (Reid and Shapley, 1992, 2002; Lee et al., 1998; Martin et al., 2001; Buzás et al., 2006; Field et al., 2010; Crook et al., 2011; Martin et al., 2011; Lee et al., 2012), and the question of the degree to which cone-type specific wiring contributes to midget receptive fields remains open.

Corresponding to the parallel pathways in the retina, color selectivities in the LGN cluster around the two cardinal axes of cone opponency (Derrington et al., 1984). In visual cortex, however, the representation of color is different. Chromatic information is encoded cortically by both opponent and non-opponent neurons (Lennie et al., 1990; Wachtler et al., 2003). Moreover, the preferences of cortical color-selective neurons are not restricted to two main axes of opponency, but are more distributed (Lennie et al., 1990), indicating a population code for color (Wachtler et al., 2003). The transformation from coding along cone opponency axes to a distributed representation in the cortex is not well understood, but according to the theory of efficient coding (Barlow, 1961, 2001) one hypothesis would be that this code is in some sense more efficient.

Previous studies investigating efficient codes for color in natural scenes have used independent component analysis (ICA), a method for finding a linear transformation that makes the resulting outputs as statistically independent as possible (Jutten and Herault, 1991; Bell and Sejnowski, 1997). Analyses of chromatic natural images using ICA revealed that opponent codes are efficient to encode natural color stimuli. Typically, in these studies two main types of chromatic selectivity were found (Hoyer and Hyvärinen, 2000; Wachtler et al., 2001; Doi et al., 2003), which qualitatively resembled more the representation in retina and LGN than the coding properties in the visual cortex. While the discrepancies can be explained in part by the stimuli used in the experiments to determine color tuning in visual cortex (Caywood et al., 2004), broadly distributed color selectivities have also been found with other types of stimuli (Wachtler et al., 2003).

A reason for the lack of insights about the nature of the distributed cortical representation of color from previous studies could be that the assumed model of a linear transformation of cone signals was not appropriate. Comparison of efficient codes found by methods like ICA with properties of neurons in the visual system requires that visual processing can be approximated as a linear transformation of the cone signals. However, the spatio-chromatic processing in the retina transforms the cone signals in fundamentally nonlinear ways and a linear model may not be adequate. Moreover, spatial center-surround filtering as observed in retinal neurons removes much of the spatial correlations between signals of neighboring cones (Wachtler et al., 2007), which may enhance the relative contribution of chromatic variation. It is further conceivable, given the limited number of fibers in the optic nerve as compared to both the number of receptors in the retina and the number of neurons in primary visual cortex, that retinal processing is subject to different constraints and coding objectives than the representation in the visual cortex (Lee et al., 2002). Under this assumption, retinal processing could be considered as a preprocessing step separate from cortical processing (Doi et al., 2003), and it would be appropriate to perform the analyses not on the cone signals, but on the output signals of the retina. Here we investigated the consequences of nonlinear spatio-chromatic filtering in the retina for the efficient coding of chromatic information in natural scenes. We modeled the center-surround processing in the retina to obtain estimates of the signals in the different parallel retinal pathways carrying chromatic information and analyzed these signals by performing ICA.

## 2. Materials and methods

### 2.1. Image basis

As image basis for the main analysis we used eight images from the hyper-spectral image database by Párraga et al. (1998) that were previously used to study efficient codes for color (Wachtler et al., 2001; Lee et al., 2002). We used the same set of images in order to make our results directly comparable with these studies. The images were recorded outdoors around Bristol, UK, under stable weather and lighting conditions (Párraga et al., 1998). In additional analyses, we used other images from the Párraga et al. dataset as well as from other datasets (see below). Images were 256 × 256 pixels in size, with each pixel subtending 0.056 × 0.056 degree of visual angle. Pixels corresponded to radiance values in 31 wavebands between 400 nm and 700 nm. Radiance values were derived from the raw data with the code provided by Párraga et al. (1998). In all scenes a Kodak GrayCard reflectance standard was present; the corresponding picture areas were ignored during analysis.

To control for potential misalignment between the color planes in the hyper-spectral images due to the relatively long acquisition time (Párraga et al., 1998), we estimated the drift between image planes by 2-d cross-correlation. In most cases, the misalignment was zero, and non-zero misalignments appeared unsystematic, with a maximum shift of 2 pixels. Repeating the analysis with images where these shifts had been corrected did not alter the findings. As an additional control we used the four images of the hyper-spectral dataset of Foster and Nascimento (Nascimento et al., 2002; Foster et al., 2006) that showed natural scenes. The individual images were recorded within 15 s, which largely excluded any misalignment of wavelength planes. The results of this analysis, as well as those of an analysis with all the images in this dataset that were larger than 600 × 600 pixels, did not change any of the findings.

### 2.2. Image filtering

**Figure 1:**
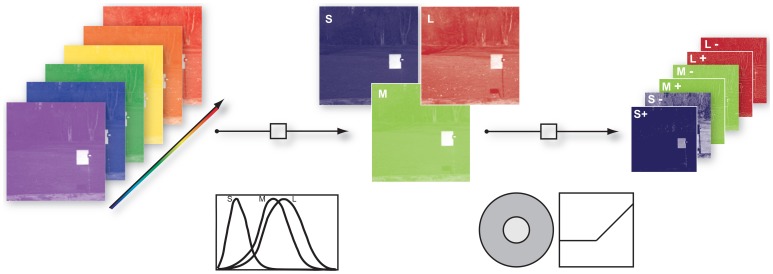
**Illustration of the image filtering process**. (1) Illustration of hyper-spectral image. Each image consisted of 31 image planes corresponding to radiance within wavebands between 400 nm and 700 nm (2) S-, M-, and L- photoreceptor cone activation were derived via their specific sensitivities. (3) S, M, and L center ON and OFF channels were obtained by convolution of the cone activations with a Mexican hat-like spatial filter, simulating the center-surround receptive fields of the retina, and splitting the result into positive and negative parts.

To take into account the spatio-chromatic filtering by the retina, three main processing steps were modeled: (a) transduction of photons to neural signals by the photoreceptors, (b) center-surround integration of cone signals, and (c) splitting of the signals in ON and OFF pathways, mediated by bipolar cells with center-surround receptive fields. Figure 1 provides an overview of the entire filtering process.

To obtain cone excitations from natural scenes, for each of the images we computed the dot products of the pixel spectra with the vectors of cone sensitivities, resulting in a 256 × 256 × 3 matrix. Human cone sensitivity estimates were taken from Stockman et al. (1993). The center-surround integration stage was modeled by convolution of the image with a Mexican hat-like kernel. We used an approximation of a Difference-of-Gaussian, consisting of a 3 × 3 pixel matrix with a value of 1 at the center, −0.15 at the center pixel of each edge, and −0.1 at the corner pixels. This filtering assumed that total weights of center and surround are balanced, and that each pixel of each pixel plane represents a ganglion cell with a single cone in the center. We assumed that the surround consists of all three cones exerting an equal contribution at each location (mixed L,M,S surround), but tested other configurations as well. ON and OFF signal channels were generated by half-wave rectification on the filter outputs and their sign-inverted counterparts, respectively. After the rectification procedure we log-transformed every channel to mimick a compressive response function. Since the rectification step introduced zero values into the data and the natural logarithm diverges at zero we added a dynamic offset to the channel. The offset was chosen such that all channels had the same dynamic range.

For the analysis, 7 × 7 patches were selected randomly from the prefiltered data. ON and OFF pixel planes for all 3 cone classes were interleaved at each pixel. The resulting dimensionality of a single input data sample was thus 7 × 7 × 3 × 2 = 294.

### 2.3. ICA

ICA was proposed as a solution to the blind source separation problem and has been applied in various studies (Bell and Sejnowski, 1997; Wachtler et al., 2001; Lewicki, 2002) to learn efficient codes for visual stimuli. The ICA model assumes a linear mixture of statistically independent sources *s* (also often called causes), which is observed via a number of sensors. If no additive sensor noise is assumed, the problem can be written as:

(1) x=As

Note that neither the sources *s* nor the mixing matrix *A* are known. The goal of ICA is to recover the sources by adapting *A* such that the resulting signals are as statistically independent as possible.

Once *A* has been inferred, the source can simply be uncovered by solving for *s*:

(2) $$s=A^{-1}x=Wx$$

The columns of *A* are usually called the basis functions and the rows of *W* are called the filters.

Here we used the approach by Lee and Lewicki (2000) with the learning rule for *A* given by:

(3) $$\Delta A\propto AA^{T}\frac{\partial }{\partial A}\text{​}\log p(x|A)=-A(z(s)s^{T}-I).$$

The individual terms are the identity matrix *I*, the transpose of the sources *s*^*T*^ and $z(s)=\frac{\partial \;\log p(s)}{\partial \;s}$. The prior source distributions were modeled using the exponential power distribution (also known as the generalized Gaussian or generalized Laplacian). The simple form is:

(4) $$p(s_{i})\propto e^{-\frac{1}{2}|s_{i}{|}^{q_{i}}}$$

The kurtosis can be controlled by varying *q*_*i*_ and thus platykurtic, leptokurtic, and Gaussian distributions can be modeled. We used $q_{i}=\frac{2}{1+\beta_{i}}$ and estimated β_*i*_ during learning. Therefore no additional assumption about the exact distribution of the sources were made a priori. As β_*i*_ becomes bigger, the distribution becomes more leptokurtic and the resulting code more sparse, meaning that the source coefficients are mostly close to zero.

The mixing matrix *A* was estimated in 100.000 iterations. It was initialized with Gaussian distributed random values and all priors were set to Gaussian densities. After every 400 iterations new input data were sampled by drawing 5000 patches randomly from each of the eight pictures and β_*i*_ was re-estimated. All samples were centered and rescaled to have zero mean and unit variance. The stepsize was adjusted at iteration points 1000, 5000, 10000, 30000, 70000, to 0.02, 0.01, 0.005, 0.002, 0.001 and 0.0001 respectively. In order to accelerate the learning process, the algorithm was ported to the CUDA parallel computing architecture and run on a NVIDIA Tesla M2090 graphics processor.

### 2.4. Analysis of the results

#### 2.4.1. Reverse correlation - activation triggered averages

After learning, the mixing matrix *A* and the unmixing matrix *W* were adapted to the preprocessed data. Due to the non-invertible nonlinear filtering, the result was not a simple linear unmixing of LMS signals. Therefore, we used a reverse-correlation approach to illustrate the resulting filters: Source activations for each post-filtered patch were used as weights for the corresponding original patch in LMS-color space. By averaging over all weighted original patches we computed the activation triggered average (ATA), i.e., the average patch that would elicit the maximal response for a single basis function. The details of this procedure are as follows:

When the individual filters *w*_*r*_ (rows of ***W***) are used to perform the unmixing of the data, each individual source coefficient *s*_*r*_ is a direct measure of the response of the filter *w*_*r*_ to a given data sample *x*_*r*_. In our case the data samples were the preprocessed patches at *p*(*i*), where the vector *i* = (*x, y, e*) specifies the patch position (*x, y*) in the preprocessed image *e*. Using the transformation between the preprocessed patches *p*_*k*_(*i*) and the patches in LMS space $\hat{p}(i)$, we can then calculate the average original patch *ATA*_*r*_ that the individual filters *w*_*r*_ best respond to by using the source coefficient derived from *p*(*i*) as a weight for $\hat{p}(i)$ and then averaging over all available $\hat{p}(i)$.

**Figure 2:**
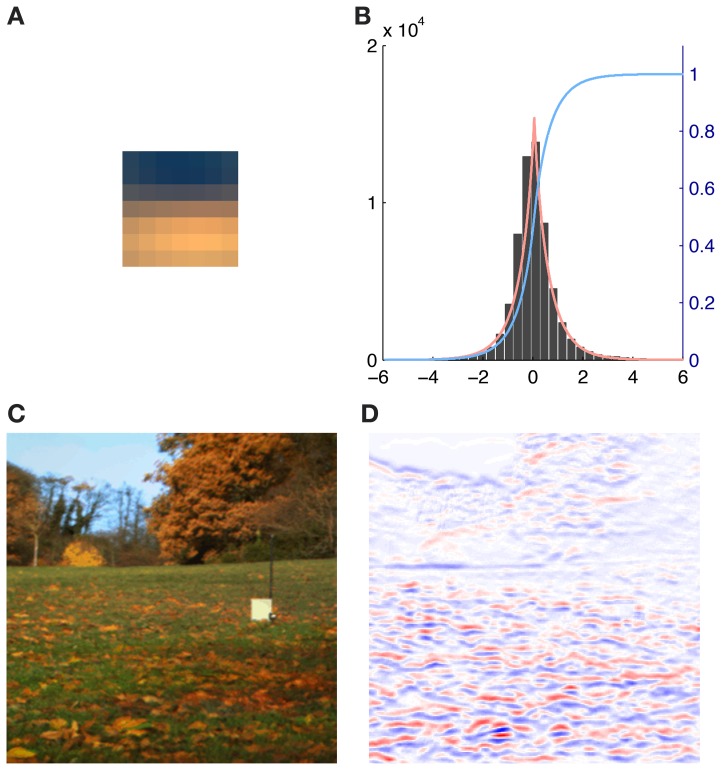
**Illustration of the contribution of a single basis function (number 116) to an image together with the exponential power distribution fit of the corresponding source activations. (A)** Activation triggered average of basis function 116. **(B)** Histogram of the source coefficient values showing a typical leptokurtic distribution with a peak around zero and heavy tails. The fit of the exponential power distribution to the data (β: 1.312, μ: 0.001, σ: 0.753) is shown in red and the cumulative distribution function *F*(*x*) is plotted in blue. **(C)** RGB rendering of the original image. **(D)** Contributions to the image, red indicates high positive, blue high negative and white no contributions of basis function 116.

We therefore generated all possible 7 × 7 patches from each of the eight preprocessed images used for analysis (*N* = 8 * 61504 = 492032). The source activations were then estimated using equation (2). To eliminate noisy contribution of source coefficients with a very low absolute activation, i.e., source activations around zero, we fitted the source activation with an exponential power distribution. When the mean of the fit was close to zero and the distribution was leptokurtic we used the cumulative distribution function *F*(*x*) to discard 95% of all the source activation around the peak (see Figure 2). Thus for each basis function and each patch in every image we computed a weight α_*r*_(*i*):

(5) $$\alpha_{r}(i)=\{\begin{array}{l} 0\text{ if }F(x)>\text{0}.\text{25}\wedge F(x)<\text{0}.\text{975}\\ s_{r}(i)\text{ otherwise} \end{array}$$

To calculate the patch in LMS-space that each basis function would maximally respond to (the ATA), we weighted each original patch $\hat{p}(i)$ with the corresponding weight α_*r*_(*i*) that was calculated earlier and averaged over the result:

(6) $$ATA_{r}=\frac{1}{N}\sum_{k=1}^{N}\alpha_{r}(k)\;\hat{p}(k)$$

We verified the plausibility of this approach by comparing the *ATA*_*r*_ with the basis functions for the analysis of LMS input without preprocessing as in Lee et al. (2002). ATAs tended to be slightly less saturated than the corresponding basis functions but otherwise they resembled each other in color preference and spatial structure almost completely.

#### 2.4.2. Plotting of the results

**Figure 3:**
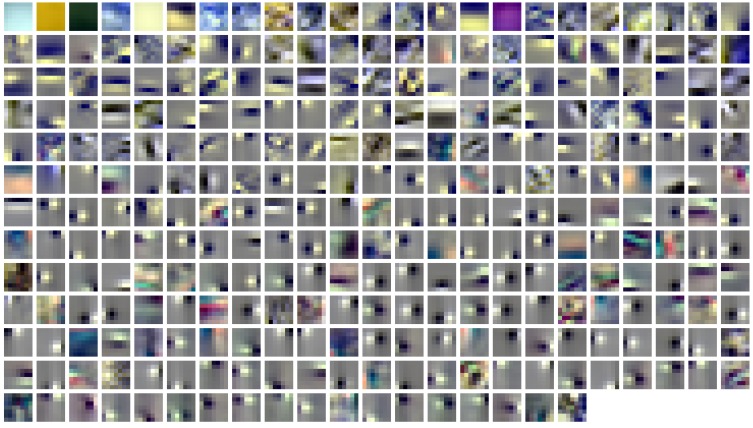
**Spatio-chromatic structure of the activation triggered averages for all 294 learned ICA basis functions**. R, G, and B pixel values for each 7x7 patch correspond to L, M, and S cone excitations that were derived from individual basis functions using a reverse correlation based approach.

We displayed the *ATA*_*r*_ as shown in Figure 3 with the method used by Ruderman et al. (1998). The L, M, and S components of each pixel of the patches were first normalized to values between 0 and 1 and then plotted as red (R), green (G) and blue (B) values. This gives a pseudo-color representation of relative cone excitations that is qualitatively similar to a true-color rendering. Therefore spatial as well as chromatic structure can be observed. To further illustrate the chromatic properties, each pixel was plotted as a point in a cone-opponent color space (Wachtler et al., 2001), where x values were computed as *x* = *L* − *M*, y values *y* = *S* − (*L* + *M*)/2 and the *z* values as *z* = *L* + *M*. When *x, y, z* values are converted to a spherical representation *r*, θ, ϕ, the azimuth angle ϕ is a direct measure of the hue of a given point while the radial distance *r* indicates its chroma and the elevation θ specifies the luminance. For plotting points we used a projection of the z-axis onto an isoluminant plane. Luminance information can still be inferred from the brightness of the individual points.

#### 2.4.3. Directions in color space

Once transferred to the cone-opponent color space, the chromatic characteristics of each patch could be quantitatively studied. To quantify the degree of opponency of individual patches, i.e., whether the pixel selectivities were roughly aligned in color space, we performed principal component analysis on the color space coordinates of all the pixels in each patch. The highest eigenvalue was used as an estimate of the strength of opponency, and the eigenvectors were used to estimate the directions of opponency. Additionally, the average color preference for a given patch was calculated by the center of mass of all points.

To quantify how uniformly a set of directions *O* were spread out in color space we calculated the Kullback-Leibler-Divergence (*D*_*KL*_) from a uniform distribution *U* with the same number of directions as $O:D_{U}(O)=D_{KL}(U∥O)=\sum_{i}ln(\frac{U(i)}{O(i)})U(i)$. The higher the value of this measure, the higher the divergence from uniformity. This measure can then be used to compare different sets of directions *O*_*n*_ derived from different ATAs.

### 2.5. Sparseness characterization

To quantify the sparseness of the resulting code and thus its efficiency, we used the criteria proposed by Willmore and Tolhurst (2001): The mean lifetime kurtosis $\bar{K_{L}}$, the population kurtosis $\bar{K_{P}}$ and the dispersal of the learned code. Both kurtosis values were computed via the standard kurtosis measure. The lifetime kurtosis *K*_*L*_ of the response, i.e the source activation of a single component is a measure of how active this component is across all stimuli. The population kurtosis *K*_*P*_ quantifies how many filters are active to encode a single stimulus. A high average *K*_*P*_-value means that only a small number of available filters are active for any given input. The dispersal of the code is a measure of the contribution of each filter to the encoding of the data. It is based on measuring the variance of the response of a filter to the image data. For a given code the standard deviation of all filters is estimated for each image and then normalized to the highest value and sorted according to their normalized standard deviation. In a compact code only a few filters encode the majority of the total variance of the data so the relative standard deviation of only a few filters will be high (close to one) and close to zero for all others. In a more dispersed code where individual filters have all higher contributions to the data, the relative standard deviation will be higher for all filters.

## 3. Results

### 3.1. Spatio-chromatic structure

**Figure 4:**
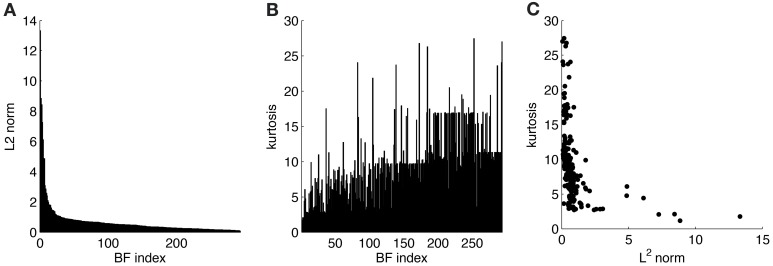
**Statistics for learned ICA basis functions. (A)** Histogram of *L*^2^ norms for every individual basis function after sorting. **(B)** Lifetime kurtosis of the source coefficients for each basis function (sorted as in **A**). **(C)** Lifetime kurtosis vs. the *L*^2^ norm.

**Figure 5:**
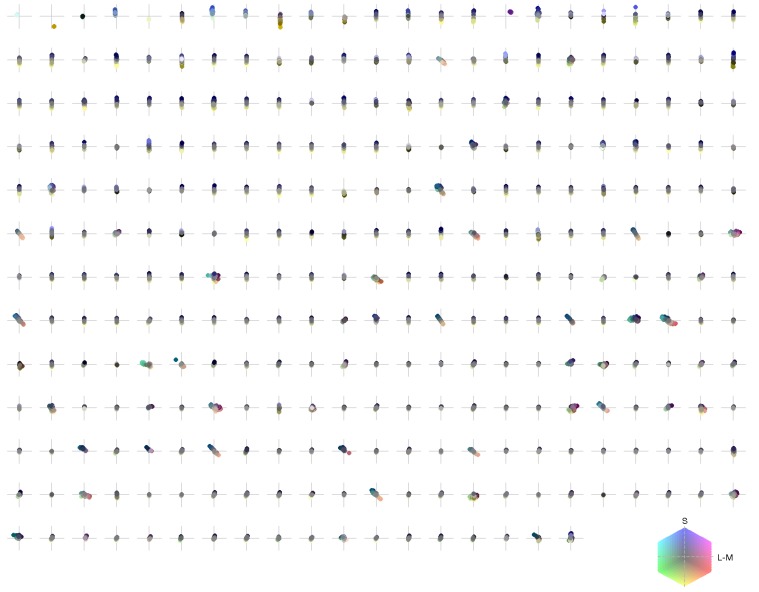
**Chromaticities of activation triggered averages: each pixel of every individual patch is projected onto an isoluminant plane in a cone-opponent color space**. The horizontal axis is defined as L − M and the vertical axis as S − (L + M)/2. Luminance values correspond to brightness of pixels.

ATAs for all 294 basis functions are shown in Figure 3. ATAs are sorted according to the *L*^2^ norm of the corresponding basis functions (see also Figure 4). The *L*^2^ norm can be used as a measure of the relative contribution of each basis function to the data. The chromaticities of the individual pixels of each ATA plotted in the cone-opponent color space are shown in Figure 5. Visual inspection of Figures 3, 5 suggests that almost all ATAs can be divided in three major categories: homogeneous chromatic, color-opponent, and achromatic. Homogeneous chromatic ATAs have a large *L*^2^ norm, no defined spatial structure, and are highly selective for one color. Most non-homogeneous but chromatic ATAs were color-opponent, i.e., the pixel chromaticities, when plotted in color space, were all clustered along a line and most often also crossed into opposing quadrants. Their spatial structure was both localized and oriented, i.e., they encoded chromatic edges (cf. Wachtler et al., 2001). A small number of non-homogenous chromatic ATAs were less strongly opponent with their pixel values more scattered in color space. There was no substantial correlation between the *L*^2^ norm of the basis function and the degree of opponency (*r* = 0.1). The achromatic ATAs, encoding luminance edges, had mid- to low-range contributions. This is a notable difference to previous findings (Wachtler et al., 2001; Lee et al., 2002), where many achromatic basis functions with high *L*^2^ norm were found (see below).

### 3.2. Distribution of color preferences

**Figure 6:**
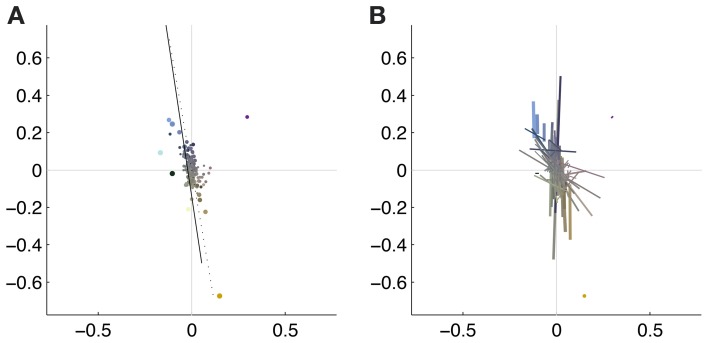
**Coverage of color space by basis functions**. The thickness of the each dot **(A)** or line **(B)** is scaled with the *L*^2^ norm of the basis function, the length of the line corresponds to the eigenvalue obtained from the PCA of the pixels chromaticities (cf. Figure 5). The position of the dots and the midpoint of the line indicate the center of gravity. Solid line: daylight axis; Dotted line: direction of first principal component of center of mass distribution.

To illustrate the overall color preferences of ATAs, we computed the center of mass of all pixels for a single ATA. The resulting positions are plotted in Figure 6A. Additionally, the direction of largest variation around the center of mass position is shown in Figure 6B. The center of mass positions were all densely clustered around the origin, indicating relatively weakly pronounced selectivities, with the exception of the homogeneous ATAs, which were more eccentric. All points together formed a distribution that was strongly elongated along a certain direction in color space. This direction, as estimated by the first principal component of the distribution, had an angle of 101.6 degrees in this color space. This matches closely the perceptual “yellow”-“blue” line, approximated by the line between the loci of monochromatic blue light of 476 nm and monochromatic yellow light of 576 nm (Mollon, 2006), which also lies close to the line of natural daylight variation and has an angle of 98.5 degrees in this color space.

**Figure 7:**
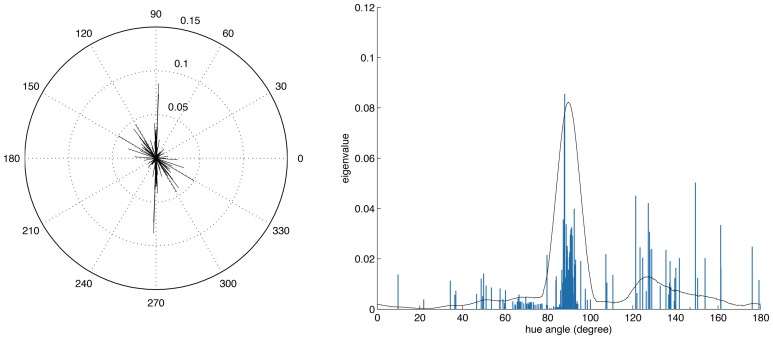
**Distributions of opponency directions in color space**. Each line in the polar and linear plot indicates the direction of the opponency axis for a single ATA or basis function. The lengths of the lines indicate the spread of pixels along the opponency axis. Since most of the color-opponency axes passed through a region very close to the origin into the opposing quadrant, we only show the range between 0 and 180 degrees in the linear plot.

Figure 7 shows the distribution of color preferences across directions in color space. In contrast to previous results obtained without spatio-chromatic preprocessing (Wachtler et al., 2001; Lee et al., 2002), color preferences were spread around the entire color space. However, the distribution was not uniform but showed several regions of higher density. One of these regions was around 90 degrees, with pixel chromaticities varying between light-blue and dark-yellow. This corresponds to a modulation of values along a plane defined by S-cone and luminance variation. Many ATAs with this chromatic signature had localized and oriented spatial features that qualitatively resembled the structure of the basis functions found for natural gray-scale images (Olshausen and Field, 1996; Bell and Sejnowski, 1997) and the achromatic basis functions for L-,M-, and S-cone activations (Wachtler et al., 2001; Lee et al., 2002). The second region with higher density was around 130 degrees, which corresponds to an opponency axis between orange and teal. These regions correspond to the two opponency axes found in previous studies (Wachtler et al., 2001; Lee et al., 2002). Another more densely covered region appeared in the first quadrant around 65–80 degrees, and the region around 10–30 degrees, was the least densely covered area. Apart from these modulations in density, the directions of color-opponent axes were widely distributed in color space with a divergence from uniformity *D*_*U*_(*O*) of 16.65, compared to the code obtained from pure LMS cone activation (Wachtler et al., 2001; Lee et al., 2002) which had a *D*_*U*_(*O*) value of 25.58.

To further analyze the chromatic properties we determined for each ATA the color tuning of the pixel with the maximal absolute value. This is comparable to estimating the color preference for small colored spots. By using this measure the directions of color preference were even more uniformly distributed in color space, with a *D*_*U*_(*O*) of 2.59 for the filtered data as compared to 21.7 for LMS data.

### 3.3. Coding efficiency

Coding efficiency was originally understood in terms of redundancy reduction. Under this assumption a code is efficient if it reduces the mutual information between components, i.e., the information encoded among a group of neurons would be reduced as much as possible. Another measure of coding efficiency especially when dealing with a large number of encoding neurons, such as in the cortex, is the sparseness of the code, i.e., how many neurons of all that are available are used to encode a specific stimulus.

A quantitative study of redundancy reduction efficacies of different linear filtering algorithms was done extensively by Eichhorn et al. (2009). Multi-information reduction, Average Log-Loss and rate-distortion curves were used as evaluation criteria for various algorithms like ICA and Principal Component Analysis (PCA), which were all compared to a random decorrelation method that served as baseline. We used the source code provided with the paper and adapted it to process our prefiltered and rectified cone signal data. Even though we kept the changes to a minimum in order to stay as close to the original analysis, it was not possible to use the NPL entropy estimator for the filtered data due to numerical instabilities. The reason for this most likely is that the distribution of the data after our preprocessing does not fit with the model assumptions of the NPL entropy estimator. Therefore, we used the Gaussian upper entropy bound (Bethge, 2006). Our results are thus not directly comparable to those in Eichhorn et al. (2009). Nevertheless the absolute multi-information reduction with respect to the random decorrelation transform (RND) was one order of magnitude better for ICA than for PCA, namely −0.4640 ± 0.0058 bits/component (ICA) and −0.0460 ± 0.0013 bits/component (PCA). The relative reduction in multi-information (cf. Table 1. in Eichhorn et al., 2009) compared to RND was 0.42 ± 0.01 percent for ICA, and 0.04 ± 0.00 percent for PCA. The Average Log-Loss (ALL), as a measure of how well the density model of the specific transformation matches the actual data and differences correspond to coding cost (Eichhorn et al., 2009). The difference for PCA-RND was −0.0481 ± 0.001 bits/component, for SSD-RND (spherically symmetric density) it was −0.2429 ± 0.0001 bits/component, and for ICA it was −0.4206 ± 0.0036 bits/component. Thus, in our case ICA performed best, i.e., it had the smallest ALL value.

**Figure 8:**
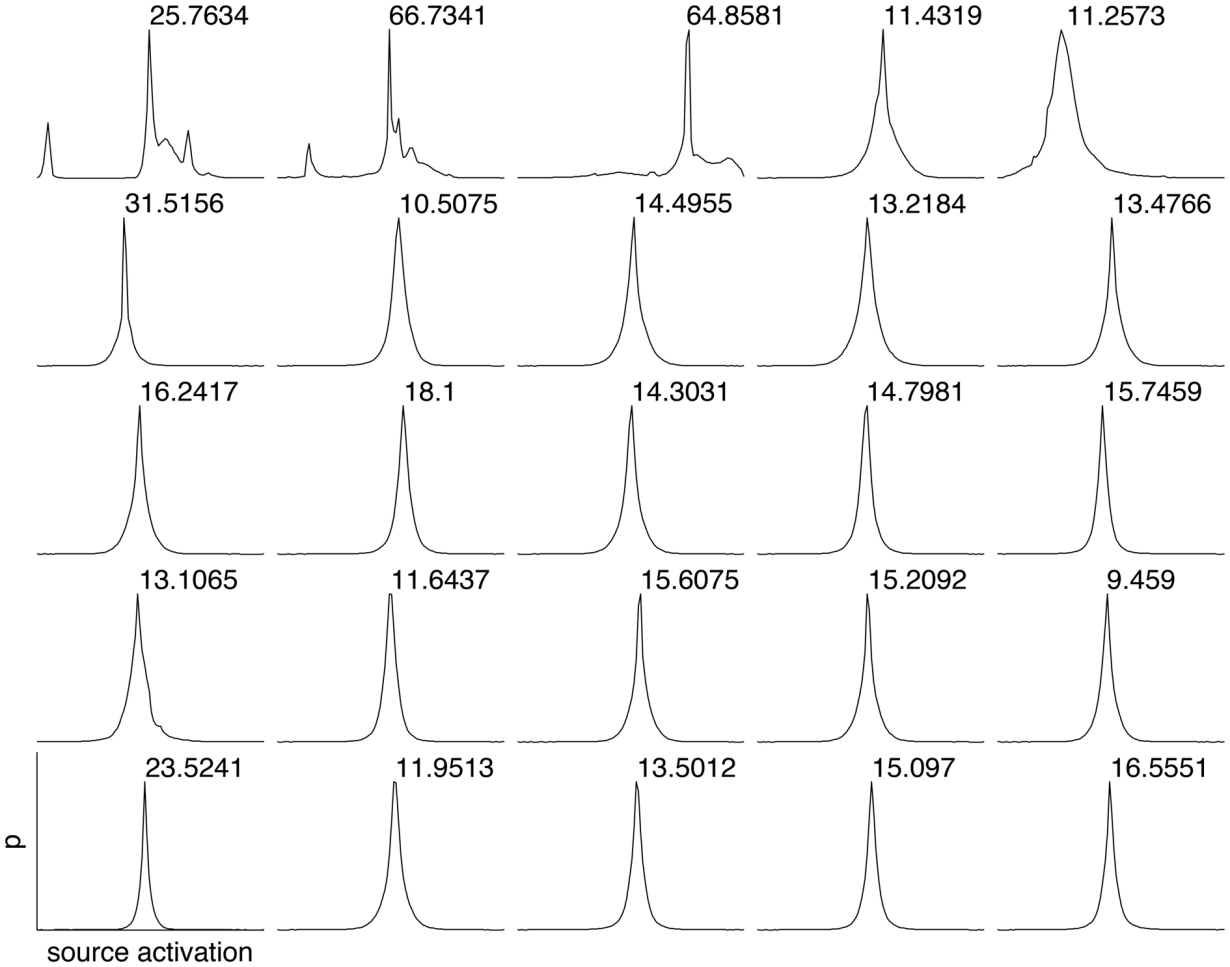
**Histograms of the source coefficient values for the first 25 basis functions and the standard kurtosis derived from the source coefficients**.

**Figure 9:**
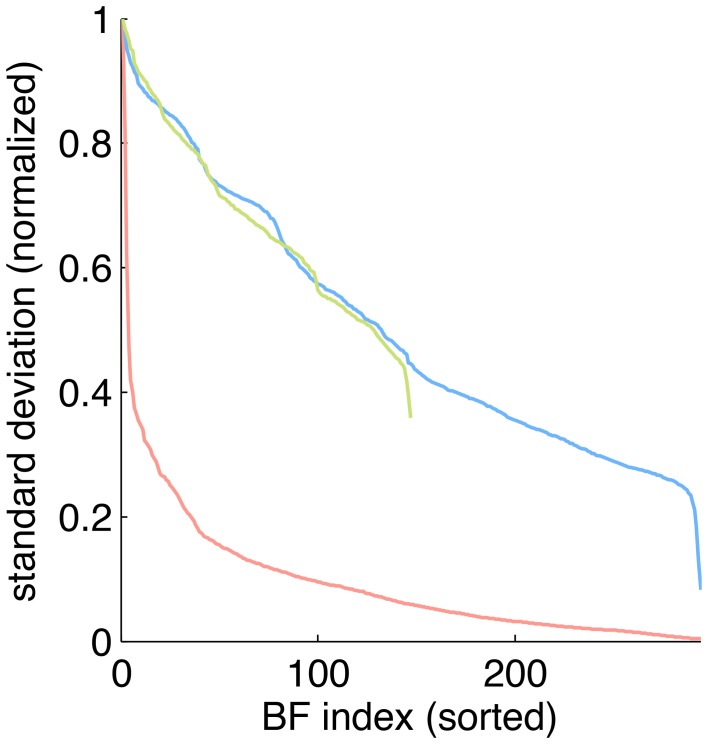
**Dispersal of different codes: ICA on preprocessed data (blue), PCA on preprocessed data (red) and ICA on LMS images (green)**. For each code, the mean of the per image response standard deviation normalized to the largest value is plotted for each basis function. Note that for unfiltered LMS images the dimensionality of the data is lower, yielding only 147 basis functions.

To estimate the sparseness of the learned representation we computed the lifetime kurtosis *K*_*L*_ of individual units and the population kurtosis *K*_*P*_ (cf. Willmore and Tolhurst, 2001). Figure 8 shows histograms for source coefficients together with their estimated lifetime kurtosis. All source densities are highly sparse (i.e., leptokurtic), with a pronounced acute peak at zero and heavy tails. The mean lifetime kurtosis $\bar{K_{L}}$ over all units was 10.29, which means individual units were silent for almost all inputs but very strongly activated for specific input features. The mean population kurtosis over all inputs was 12.67, i.e., only a small subset of available neurons were active for any given input. In addition to lifeline and population kurtosis, the dispersal is an important measurement for the sparseness of the code. This measure, based on the standard deviation of the responses, quantifies the relative coding contribution of each filter (derived from the basis function) to the image data. Figure 9 shows the dispersal for ICA, and as a comparison for PCA, which is an example of a compact code. PCA was done on the same preprocessed cone activation data. For ICA the decrease in coding contribution is close to linear, while for PCA it is exponential. This confirms that PCA is a much more compact code, i.e., only a few filters are used to encode the majority of the data, while in the ICA case most of the filters have a high contribution. Overall, these results show that, in accordance with previous studies (Lee et al., 2002), the obtained representation for the preprocessed images was a highly disperse and sparse, i.e., statistically efficient, code, although sparseness itself was not an enforced constraint during learning.

## 4. Discussion

We investigated the consequences of nonlinear spatio-chromatic filtering similar to the processing in the retina, including the splitting into parallel ON and OFF color-opponent channels, for the learning of efficient codes from responses to natural scenes. Compared to the results of previous studies where ICA was used to learn efficient codes directly from LMS cone activations of natural images (Wachtler et al., 2001; Lee et al., 2002), chromatic preferences obtained from opponent signals were more broadly distributed in color space. A continuous distribution is in better accordance with experimental data (Lennie et al., 1990; Wachtler et al., 2003) than the strong clustering into three chromatic types observed previously. Additionally, it is also in closer correspondence with precortical encoding of color. The filtering we applied mimicks the effect of center-surround receptive fields of retinal bipolar and ganglion cells, which removes redundancy both in the spatial and the spectral domain. In previous studies, whitening had been applied in a linear preprocessing stage before ICA. However, to estimate the results, this pre-filtering had to be taken into account by adding a corresponding linear transformation. In our analysis, such a direct compensation would not have been possible because the preprocessing stage was a nonlinear transformation. To represent the resulting components, we therefore used a reverse correlation technique to obtain a single-stage linear transformation representing the effective linear component of the multi-stage nonlinear filtering. A further effect of the preprocessing was the representation of the signals in the higher-dimensional space of six rectified opponent channels. This representation may have facilitated the distinction of features (Schölkopf and Smola, 2002). Similarly, the parallel channels in the retina and LGN provide such a high-dimensional representation, which might be exploited by cortical learning mechanisms.

The set of natural images was chosen to be the same as in Wachtler et al. (2001); Lee et al. (2002) to enable direct comparison of the results. These images were initially chosen to include a variety of scenes recorded outdoors and under different illuminations. To exclude that our results were an artifact of the specific choice of images, we repeated the analysis including all outdoor images contained in the Párraga et al. (1998) dataset. The result was again a broad distribution of chromatic preferences with a divergence from uniformity of 15.91, compared to a value of 28.64 obtained for these images without pre-filtering. In addition, we ran an analysis using a larger patch size of 10×10 pixel. This resulted in a spread of selectivities that was even more broad, with a divergence from uniformity of 11.09 compared to 24.27 obtained without pre-filtering.

For the center-surround spatio-chromatic filtering we used filters with a surround composed of equal contributions of all cone types. Often, the center-surround processing in the retina is likened to a whitening stage that removes second-order dependencies (Doi et al., 2003). Whitening filters for LMS images typically have also a cone-type specific center-surround structure. We repeated the analysis using whitening filters for the preprocessing. The color preferences of the resulting ICA ATAs were strongly clustered around a single region in color space, which is not in line with the observed color preferences in the visual system.

Our spatio-chromatic prefiltering mimicked the opponency of small bistratified ganglion cells and of midget cells under the assumption of a cone-type unspecific wiring of the surround. However, the exact composition of the surround of retinal receptive fields is unclear (Reid and Shapley, 1992, 2002; Lee et al., 1998, 2012; Martin et al., 2001; Buzás et al., 2006; Field et al., 2010; Crook et al., 2011; Martin et al., 2011). We determined how different surround compositions affect the distributions of chromatic preferences and the sparseness of the coding. We repeated the analysis with the same parameters but with different surround structures in the filtering stage. Besides the unspecific, mixed LMS surround we also used an unspecific mixed LM surround, a cone-type specific surround, and intermediate (mixed but biased) models for the surround (cf. Table 1). In addition we also used spatio-chromatic decorrelation via Zero-phase Whitening (Bell and Sejnowski, 1997). Compared to whitening, plausible retinal filtering led to more uniform distributions. Among the considered variants of retinal filtering, an equally balanced mixed surround with contributions from all cone types resulted in the smallest deviation from uniformity, but the other surround structures yielded similar values (cf. Table 1). Our results therefore do not provide a strong indication in favor of any specific surround organization. This suggests that in the real visual system there might be a high variation in the surround composition, which could explain why experimental evidence on the specificity of the surround has so far not been conclusive.

**Table 1:** **Kullback-Leibler-Divergence from uniformity, mean lifetime kurtosis $\bar{K_{L}}$ and mean population kurtosis $\bar{K_{P}}$ for different surround configurations and preprocessing methods**.

**Surround organization**	**Surround for center cone of type**			***D_u(O)_***	$\bar{K_{L}}$	$\bar{K_{P}}$
	**L-center**	**M-center**	**S-center**			
Mixed LMS		$\frac{1}{3}(L+M+S)$		16.65	10.29	12.67
Mixed LM		$\frac{1}{2}(L+M)$		16.81	9.09	10.35
Specific	M	L	$\frac{1}{2}(M+S)$	17.70	9.25	10.80
Biased LM	$\frac{1}{2}(\frac{1}{2}(L+M)+L)$	$\frac{1}{2}(\frac{1}{2}(L+M)+M)$	$\frac{1}{2}(\frac{1}{2}(L+M)+S)$	20.23	8.86	10.04
Biased LMS	$\frac{1}{2}(\frac{1}{3}(L+M+S)+L)$	$\frac{1}{2}(\frac{1}{3}(L+M+S)+M)$	$\frac{1}{2}(\frac{1}{3}(L+M+S)+S)$	21.27	9.40	10.37
Whitening	Spatio-chromatic whitening via ZCA			32.11	9.40	4.86
None	No pre-filtering and rectification (pure L,M,S signals)			25.58	21.40	4.84

Rows 1–5 show results when surround configurations were altered while holding all other parameters constant. Additionally the results are shown for when spatio-chromatic filtering via zero-phase whitening (Bell and Sejnowski, 1997) was performed instead of center-surround filtering (row six). The last row shows the results obtained from non-preprocessed pure LMS signals as in Lee et al. (2002).

In addition to having more distributed preferences, the learned code for preprocessed data had all attributes one would expect from a sparse code in the cortex. The lifetime sparseness of individual components was high, but lower than in the case of unfiltered LMS data (10.29 vs. 21.40). On the other hand, the population kurtosis was drastically increased (12.67 vs. 4.86), meaning that only a small subset of all available units were active at the same time. This fits very well with the vast increase in number of neurons from LGN to visual cortex, which is paralleled in our study by the increase in dimensionality. Moreover, the code revealed by our analysis was also highly disperse, i.e., for different stimuli different subsets of units were active. This is in contrast to a compact code like PCA, where also only a few components are active all the time, but it is always the same components that take part in the coding. Such an unequal distribution of activity would seem biologically implausible because a majority of the neurons would be there without making substantial contributions to the encoding of the stimuli.

A substantial amount of ATAs (14.3%) had chromatic selectivities that corresponded to variation between light-blue and dark-yellow. Moreover, the overall distribution of color preferences also varied along one main direction in color space. Both of these axes of variation were very close to the perceptual blue-yellow axis and the line of variation of natural daylight illuminations (Mollon, 2006), which constitutes the main chromatic variation of natural scenes (Webster and Mollon, 1997) and was found in previous ICA analyses (Wachtler et al., 2001). It it also reflected in the peak of the distribution of color preferences in primary visual cortex (Wachtler et al., 2003). Our results support the conclusion that the statistics of natural scenes are an important factor in shaping the processing mechanisms of the visual system.

### Conflict of interest statement

The authors declare that the research was conducted in the absence of any commercial or financial relationships that could be construed as a potential conflict of interest.
